# Mechanisms of left ventricular systolic dysfunction in light chain amyloidosis: a multiparametric cardiac MRI study

**DOI:** 10.3389/fcvm.2024.1371810

**Published:** 2024-05-30

**Authors:** Ethan Katznelson, Michael Jerosch-Herold, Sarah A. M. Cuddy, Olivier F. Clerc, Dominik C. Benz, Alexandra Taylor, Shivani Rao, Marie Foley Kijewski, Ronglih Liao, Heather Landau, Andrew J. Yee, Frederick L. Ruberg, Marcelo F. Di Carli, Rodney H. Falk, Raymond Y. Kwong, Sharmila Dorbala

**Affiliations:** ^1^ Department of Cardiology, Weill Cornell Medical Center, New York, NY, United States; ^2^ CV Imaging Program, Cardiovascular Division and Department of Radiology, Brigham and Women’s Hospital, Boston, MA, United States; ^3^ Amyloidosis Program, Division of Cardiology, Department of Medicine, Brigham and Women’s Hospital, Boston, MA, United States; ^4^ Division of Nuclear Medicine, Department of Radiology, Brigham and Women’s Hospital, Boston, MA, United States; ^5^ Amyloidosis Program, Stanford University, Stanford, CA, United States; ^6^ Division of Medical Oncology, Memorial Sloan Kettering Medical Center, New York, NY, United States; ^7^ Division of Hematology and Oncology, Department of Medicine, Massachusetts General Hospital, Boston, MA, United States; ^8^ Section of Cardiovascular Medicine, Department of Medicine, Boston Medical Center, Amyloidosis Center, Boston University Chobanian & Avedisian School of Medicine, Boston, MA, United States

**Keywords:** cardiac amyloidosis, myocardial work efficiency, myocardial blood flow, extracellular volume, cardiac MRI (CMR)

## Abstract

**Background:**

Cardiac systolic dysfunction is a poor prognostic marker in light-chain (AL) cardiomyopathy, a primary interstitial disorder; however, its pathogenesis is poorly understood.

**Purpose:**

This study aims to analyze the effects of extracellular volume (ECV) expansion, a surrogate marker of amyloid burden on myocardial blood flow (MBF), myocardial work efficiency (MWE), and left ventricular (LV) systolic dysfunction in AL amyloidosis.

**Methods:**

Subjects with biopsy-proven AL amyloidosis were prospectively enrolled (April 2016–June 2021; Clinicaltrials.gov ID NCT02641145) and underwent cardiac magnetic resonance imaging (MRI) to quantify rest MBF by perfusion imaging, LV ejection fraction (LVEF) by cine MRI, and ECV by pre- and post-contrast T1 mapping. The MWE was estimated as external cardiac work from the stroke volume and mean arterial pressure normalized to the LV myocardial mass.

**Results:**

Rest MBF in 92 subjects (62 ± 8 years, 52 men) with AL amyloidosis averaged 0.87 ± 0.21 ml/min/g and correlated with MWE (*r* = 0.42; *p* < 0.001). Rest MBF was similarly low in subjects with sustained hematologic remission after successful AL amyloidosis therapy (*n* = 21), as in those with recently diagnosed AL amyloidosis. Both MBF and MWE decreased by ECV tertile (*p* < 0.01 for linear trends). The association of ECV with MWE comprised a direct effect (84% of the total effect; *p* < 0.001) on MWE from adverse interstitial remodeling assessed by ECV and an indirect effect (16% of the total effect; *p* < 0.001) mediated by MBF. There was a significant base-to-apex gradient of rest MBF in subjects with higher amyloid burden.

**Conclusions:**

In AL amyloidosis, both MBF and MWE decrease as cardiac amyloid burden and ECV expansion increase. Both structural and vascular changes from ECV expansion and myocardial amyloid burden appear to contribute to lower MWE.

## Introduction

Light-chain amyloidosis (AL) is a clonal plasma cell disorder characterized by abnormally high circulating levels of immunoglobulin fragments that misfold and deposit as fibrils in the extracellular space (1). Myocardial AL amyloid deposits are recognized as a major cause of heart failure symptoms, progression, and death. Left ventricular (LV) systolic function is typically preserved in the early stages and declines in the later stages of AL amyloidosis, which is an independent predictor of poor survival (2). The reasons for reduced LVEF in AL amyloidosis, a primary interstitial disease, remain unknown. Potential causes of myocardial dysfunction and impaired myocardial work efficiency (MWE) in AL cardiomyopathy (AL-CMP) include abnormal interstitial remodeling, reduced myocardial perfusion, and light-chain toxicity.

Myocardial blood flow (MBF) can be reduced from interstitial remodeling due to AL deposits (3), which can reduce capillary density (4), increase coronary vascular resistance (5), and cause intramyocardial coronary obstructive and non-obstructive arterial deposits (3). Reduced rest (6, 7) and stress MBF, independent of obstructive epicardial coronary artery disease, are notable features of AL-CMP (7). Additionally, infusion of amyloidogenic light chains into isolated cardiomyocytes can induce apoptosis (8), abnormal mitophagy (8), and contractile dysfunction, collectively termed as light-chain toxicity (9). Therefore, reduced MBF from severe interstitial and coronary arterial remodeling and cardiomyocyte abnormalities, including deranged myocardial metabolism and energy efficiency, are plausible mechanisms of LV systolic dysfunction in AL-CMP. Previous studies have not fully evaluated the pathogenesis of LV systolic dysfunction in AL-CMP.

Cardiac magnetic resonance imaging (MRI) enables an integrated evaluation of regional myocardial function, absolute MBF, and extracellular volume (ECV) expansion indicative of amyloid deposition through contrast-enhanced T1 mapping and myocardial tissue characterization. Although not exclusively resulting from amyloid deposition, ECV expansion provides valuable insights into the myocardial response to amyloid deposition.

To evaluate the prevalence and degree of myocardial dysfunction due to amyloid deposition, we assessed herein MWE. We hypothesized that MWE would be reduced in subjects with AL amyloidosis due to adverse interstitial remodeling, light-chain toxicity, and reduced rest MBF. The objective of this study was to analyze the relationship between extracellular matrix expansion, MBF, MWE, and systolic dysfunction assessed by cardiac MRI (CMR) in subjects with AL amyloidosis. Furthermore, we aimed to investigate the variability of MBF within the LV and identify the relationship between regional patterns of ECV within the apical, middle, and basal regions of the LV.

## Methods

### Subject selection

This study cohort included subjects with biopsy-proven systemic AL amyloidosis who were consecutively and prospectively enrolled in the ongoing study titled “Molecular Imaging of Primary Amyloid Cardiomyopathy.” The study was approved by the Mass General Brigham Human Research Committee, and each subject provided written informed consent for participation. The subjects were recruited from five centers, namely, Brigham and Women's Hospital; Dana–Farber Cancer Institute; Massachusetts General Hospital; Boston Medical Center/Boston University School of Medicine, Boston; and Memorial Sloan Kettering Cancer Center, New York.

### Study cohort and procedures

The study cohort included subjects with systemic AL amyloidosis predominantly diagnosed by biopsy (biopsy was inadequate for diagnosis in 2.2% of cases due to insufficient quality/quantity). Amyloid typing was performed using either immunohistochemistry or mass spectroscopy. The subjects were enrolled from April 2016 to June 2021 and provided written informed consent. Those with an estimated glomerular filtration rate of <30 ml/gm/min or contraindications for MRI were excluded. All subjects underwent a detailed evaluation with serum biomarkers, gadolinium contrast-enhanced CMR, and echocardiography. MRI data were analyzed by EK and MJH. Out of the 110 subjects enrolled, 92 were included in this analysis. The reasons for exclusion included known obstructive epicardial disease and limited perfusion scans due to image artifacts or excessive breathing motion that caused shifts in slice locations.

### Cardiac imaging

#### CMR

CMR images were acquired at a single institution on a single 3.0T system (Tim Trio, Siemens, Erlangen, Germany), with electrocardiographic gating and breath holding for cine imaging with steady-state free-precession, late gadolinium enhancement (LGE) imaging, and modified Look–Locker T1 mapping. T1 mapping was performed with a modified Look–Locker technique with a 5-3-3 acquisition scheme for pre- and post-contrast mapping. Post-contrast T1 maps were acquired at 10 and 20 min after injecting a total of 0.1 mmol/kg of gadoterate meglumine contrast agent (Dotarem, Guerbet LLC, Princeton, NJ, USA). A commercial software package (MedisSuite 3.0 Medical Imaging Systems, Leiden, The Netherlands) was used for post-processing and quantification of LV volumes, LVEF, and LV mass (10). Measures of LV wall thickness, T1, and ECV were obtained for 16 LV segments in accordance with a modified AHA 17-segment model, which excluded the apical cap (11). The myocardial partition coefficient for gadolinium contrast (λ_Gd_) was estimated for each myocardial segment by linear regression of the average R1 in the segment against R1 in blood, where *λ*_Gd_ corresponds to the slope of the regression line. This linear regression method results in a lower variance of ECV estimates compared to the two-point estimate (12). ECV was calculated as (1−Hct) multiplied by *λ*_Gd_, where Hct represents the blood hematocrit of a blood sample obtained within an hour of the MRI scan. Subsequently, LGE imaging was performed with an IR-prepared single-shot gradient-echo sequence with steady-state free precession. The time after inversion (TI) was set based on the images from a TI “scout” cine sequence. The LGE images were assessed for the presence of any LGE in the LV.

The global myocardial ECV was calculated by averaging the myocardial segmental ECV values from the short-axis slices at the base, middle, and apical LV levels. LGE was not quantified due to difficulties with quantification and was therefore visually graded as present or absent. Cardiac output was obtained by multiplying CMR stroke volume by heart rate.

#### MWE

The external cardiac work (EW) in mm Hg/L per minute was calculated as the product of cardiac output and mean arterial blood pressure, as previously described (6). MWE was obtained by dividing EW by LV mass (6). MWE was previously shown to be closely related to myocardial oxygen extraction efficiency, and both measures reflected a reduced myocardial external efficiency in cardiac amyloidosis subjects (6). The conventional metric for myocardial efficiency includes measures of energy expenditure assessed by C11-acetate PET via measurement of myocardial tracer washout rate (13). However, in cardiac amyloidosis, Clemmensen et al. (6) validated this surrogate MWE as a simple alternate to myocardial external efficiency (the ratio of LV external stroke work and the energy equivalent of myocardial oxygen consumption) assessment, which does not require C11-acetate measurements. In this manuscript, the abbreviation MWE refers to surrogate MWE derived from CMR-measured cardiac output and LV mass.

#### Cardiac perfusion imaging: during the first pass of an injected contrast bolus

Myocardial perfusion was assessed by ECG-triggered, multi-slice, saturation-recovery prepared (T1-weighted), single-shot gradient-echo imaging. A dosage of 0.05 mmol/kg of gadoterate meglumine (Dotarem, Guerbet LLC, Princeton, NJ, USA) was injected at a rate of 4 ml/s during the first pass perfusion scan at approximately 4–5 s after the start of the sequence. The sequence used a linear phase-encoding ordering and a T-SENSE spatial–temporal under-sampling scheme (×2 acceleration). The other parameters were echo time (TE = 0.95 ms, TR = 2.05 ms, flip angle = 18°) and acquisition matrix (192 × 146, 8 mm slice thickness, three short-axis slices per heartbeat). The effective pixel resolution was 2 mm (i.e., without interpolation). The subject was instructed to hold breath for approximately 5 s from the start of the injection and as long as comfortable to avoid large diaphragm excursions at the end of the breath-hold. The image reconstruction on the scanner included an in-plane motion correction (MOCO). Immediately after the rest perfusion scan, a “top-off” dosage of 0.05 mmol/kg was injected for post-contrast T1 mapping at 10 and 20 min later with a total injected contrast dosage of 0.1 mmol/kg.

#### MBF quantification

The perfusion images were analyzed with the QMass CMR software (Medis, Leiden, The Netherlands) by segmenting each image along the endo- and epicardial borders of the LV. The signal intensities were averaged within each of the 16 myocardial segments (modified AHA segmentation model) to generate segmental signal intensity vs. time curves. A region of interest in the center of the LV captured the arterial input of contrast enhancement. The signal intensity values for all segments and the arterial input were converted into relaxation rate (R1) values using a model-based approach and the measured pre-contrast (native) T1 of tissue and blood (14). The MBF was quantified from the R1 vs. time curves by a validated method based on model-independent deconvolution of the myocardial tissue curves with the arterial input (15).

#### Echocardiography

2D echocardiography with spectral and color Doppler imaging was performed in all subjects according to standard American Society of Echocardiography recommendations (16). The global longitudinal strain (GLS) was derived using the Image Arena software (TomTec Imaging Systems GmbH, Germany).

### Statistical analysis

The results for the continuous variables were expressed as mean ± SD or median and interquartile range and as counts (percentage) for the categorical variables. Comparisons of the continuous variables between groups were performed with unpaired Student's *t*-test, and when involving multiple pairwise comparisons (e.g., *post hoc* pairwise tests between groups after ANOVA), the *p*-values were corrected by Holm's method. The assumption of normality was checked visually by quantile–quantile plots and by formal testing using the Shapiro–Wilk test. The pairwise correlations between variables were assessed by Pearson's method. The original study was powered to detect 20% differences in ECV. With a similar criterion for detecting a 20% difference in rest MBF, which averages approximately 1.0 ml/min/g with low amyloid burden or in healthy persons, and a typical standard deviation of rest MBF of 0.2 ml/min/g, we estimated the statistical power to be greater than 0. 9 with *N* = 30 patients per group to detect a 0.2 ml/min/g difference.

Parsimonious multivariable regression models for MWE, LV EF, and GLS were built by using the Akaike information criterion (AIC) in a stepwise algorithm (“stepAIC” in the R “MASS” package) to determine predictors from a pool encompassing ECV, rest MBF, left atrial (LA) volume index, LV mass index, age, and sex. Age and sex were forced to be in the final multivariable regression models. The mediation analysis was based on the hypothesis that the effect of ECV on MWE comprised a component directly related to adverse interstitial remodeling and light-chain toxicity and an indirect effect mediated by MBF, a key determinant of myocardial oxygen supply, myocardial external efficiency, and, by extension, MWE (6). The mediation model was built with the “lavaan” package (https://cran.r-project.org/web/packages/lavaan/index.html) for structural equation modeling, with confidence intervals for the coefficient estimates generated by the bootstrap method. The residual root mean square error of approximation (RMSEA), a fit statistic of the average of standardized residuals between the observed and hypothesized covariances, was used to assess fit quality. An RMSEA value smaller than 0.05 was considered to indicate good convergence of the fit to the data. Linear mixed-effects models were built with the “lme4” package in R. The relation between segmental MBF and the segmental ECV was analyzed with a linear mixed-effects model that included segmental ECV and the rate–pressure product (RPP) as fixed-effects predictors and a random intercept stratified by a patient, slice-level, and wall-segment location (septum vs. free wall). A second model was used to test for any systematic variation of rest MBF from the base to the apex by specifying the slice location as a fixed effect and an intercept term per subject as a random effect to account for unobserved subject-specific factors. All statistical analyses were performed with the R program (version 4.1.1; https://www.R-project.org/).

## Results

### Baseline characteristics

The baseline characteristics of the 92 subjects (mean age, 62 ± 8 years; 52 men) stratified by ECV tertiles are summarized in Table 1. Out of the 75 subjects with elevated biomarkers, 21 (23%) were in sustained hematologic remission at the time of CMR. There were no significant differences observed in age, gender, or body mass index between the three groups.

**Table 1:** Demographic characteristics of all subjects stratified by the extracellular volume fraction, a surrogate marker for cardiac amyloid burden.

Characteristic	All subjects	Stratified by ECV tertiles (*N* = 89 with ECV)			*p*-value^b^
	*N* = 92^a^	Lower (22.6, 42.3) *N* = 27^a^	Middle (42.3, 51.2) *N* = 32^a^	Upper (51.2, 68) *N* = 30^a^	
AL status					0.2
AL amyloidosis (recent diagnosis)	71 (77%)	23 (85%)	21 (66%)	24 (80%)	
AL amyloidosis (AL remission)	21 (23%)	4 (15%)	11 (34%)	6 (20%)	
Age (years)	62 (57, 68)	61 (58, 66)	62 (57, 68)	64 (57, 69)	0.8
Male sex	52 (57%)	18 (67%)	20 (62%)	14 (47%)	0.3
NYHA class					0.012
1	19 (22%)	10 (38%)	5 (17%)	3 (11%)	
2	37 (43%)	11 (42%)	13 (43%)	12 (43%)	
3	25 (29%)	2 (7.7%)	12 (40%)	11 (39%)	
4	6 (6.9%)	3 (12%)	0 (0%)	2 (7.1%)	
Height (m)	67.2 ± 3.9	68.7 ± 3.9	67.1 ± 3.6	66.4 ± 4.0	0.080
BMI (kg/m^2^)	26.1 ± 4.3	25.8 ± 4.2	26.6 ± 3.9	26.0 ± 5.0	0.8
BSA (m^2^)	1.87 ± 0.22	1.93 ± 0.21	1.88 ± 0.20	1.83 ± 0.24	0.3
Systolic BP (mmHg)	117 ± 18	124 ± 20	114 ± 18	113 ± 15	0.049
Diastolic BP (mmHg)	69 ± 12	71 ± 14	68 ± 12	69 ± 10	0.8
Troponin-T (ng/ml)	0.03 (0.01, 0.08)	0.01 (0.01, 0.03)	0.05 (0.02, 0.10)	0.04 (0.01, 0.09)	0.004
NT-proBNP (pg/ml)	1,530 (625, 4,954)	248 (88, 737)	2,607 (1,121, 6,427)	2,756 (1,715, 5,946)	<0.001
Peak LV GLS	−16.1 ± 5.0	−20.4 ± 4.7	−14.7 ± 4.2	−14.1 ± 4.0	<0.001

BP, blood pressure; BMI, body mass index; BSA, body surface area; GLS, global longitudinal strain; LV, left ventricular; NYHA, New York Heart Association; NT-proBNP, N-terminal pro-B-type natriuretic peptide.

^a^ *n* (%); median (IQR); mean ± SD.

^b^ Fisher's exact test; Kruskal–Wallis rank sum test; Pearson's chi-squared test; one-way ANOVA.

### ECV and MBF

**Figure 1:**
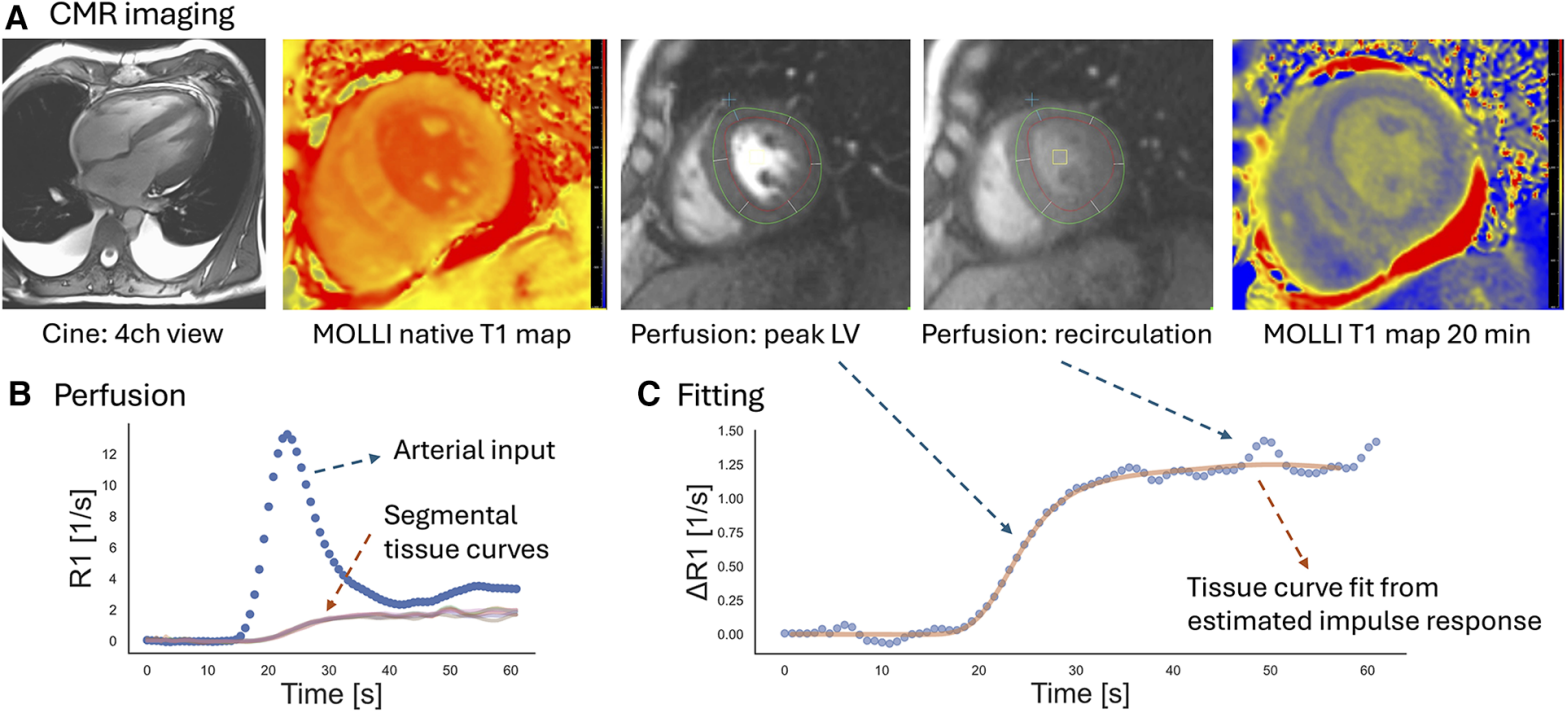
Example of the CMR assessment of perfusion and T1 mapping in a patient with AL-CMP. (**A**) For perfusion imaging at rest, three short-axis slices were imaged at every heartbeat during the first pass of a gadolinium contrast agent. Perfusion images are shown for the mid-LV level slice. T1 mapping was performed before contrast injection (“native T1”) and at 10 and 20 min after injection of the contrast agent at slice locations matching the perfusion assessment. Cine imaging of the LV indicated mild LV hypertrophy (mid-slice LV wall thickness = 15 mm). (**B**) Endo- and epicardial contours were drawn in motion-corrected first-pass perfusion images (red and green contours in the perfusion images above) to generate signal–intensity vs. time curves for a region in the blood pool and myocardial segments. The signal intensity curves were converted into curves showing the change of R1 with time. (**C**) Myocardial R1 vs. time curves were fit with the arterial input measured at the basal level by model-independent deconvolution. The solid curve shows the myocardial response calculated from the estimated impulse response and the measured arterial input. Based on Ziegler's central volume principle, the amplitude of the impulse response estimates the myocardial blood flow.

**Figure 2:**
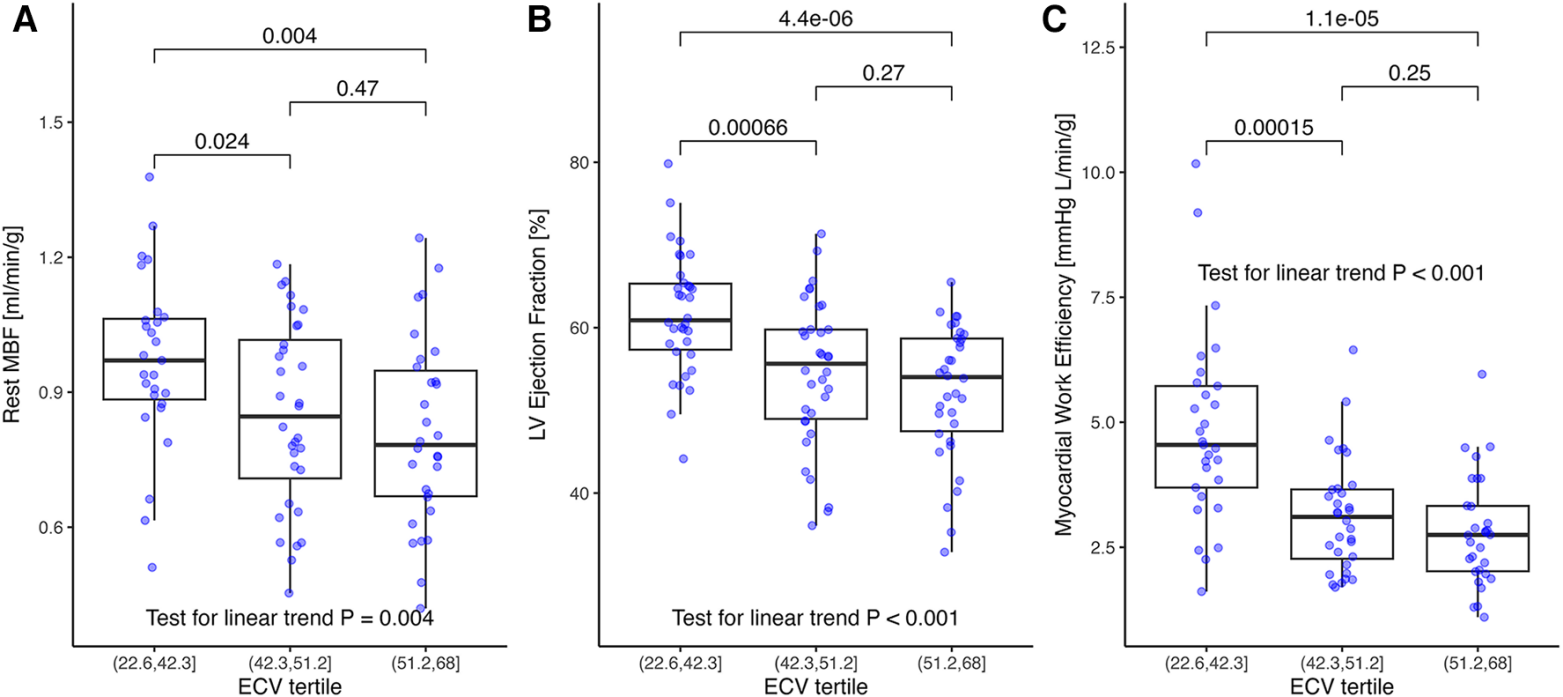
(**A**) Rest myocardial blood flow, (**B**) rest left ventricular ejection fraction, and (**C**) myocardial work efficiency stratified by ECV tertiles. (**A**) Myocardial blood flow decreased by tertiles of increasing ECV (*p* = 0.004 for linear trend). (**B**) Similarly, LVEF decreased with ECV (*p* < 0.001 for linear trend) and (**C**) for myocardial work efficiency defined as external work (stroke volume×mean arterial pressure) divided by left ventricular mass. The *p*-values in brackets are from the *t*-tests and adjusted for multiple comparisons. ECV, extracellular volume; LVEF, left ventricular ejection fraction.

Figure 1 provides examples of the perfusion assessment and T1 mapping in one patient with AL-CMP. The ECV averaged 46% ± 10% and was categorized into tertiles to reflect the varying degrees of disease-related extracellular matrix expansion. The MRI-related measurements stratified by ECV tertiles are summarized in Table 2. MBF averaged 0.87 ± 0.21 ml/min/g and was moderately correlated with the RPP (*r* = 0.36, *p* < 0.001), a measure of cardiac workload. MBF was decreased by ECV tertiles, as shown in Figure 2A (*p* = 0.004 for linear trend). There was no significant linear trend for MBF normalized by RPP across ECV tertiles.

**Table 2:** Summary of the cardiac MRI imaging findings for all subjects stratified by tertiles of extracellular volume.

Characteristic	All subjects	Stratified by ECV tertiles (*N* = 89 with ECV)			
	*N* = 92^a^	Lower (22.6, 42.3], *N* = 27^a^	Middle (42.3, 51.2], *N* = 32^a^	Upper (51.2, 68], *N* = 30^a^	*p*-value^b^
Heart rate (bpm)	77 ± 12	76 ± 12	78 ± 12	76 ± 13	0.8
RPP (sys. BP x bpm/1e4)	0.90 ± 0.21	0.95 ± 0.25	0.88 ± 0.17	0.87 ± 0.20	0.3
LV mass index (g/m^2^)	80 ± 27	64 ± 21	83 ± 20	93 ± 32	<0.001
LV EDV index (ml/m^2^)	71 ± 15	69 ± 13	69 ± 13	76 ± 17	0.11
LV ESV index (ml/m^2^)	32 ± 12	27 ± 8	32 ± 11	37 ± 14	0.006
LVEF (%)	56 ± 9	61 ± 7	54 ± 9	52 ± 8	<0.001
Cardiac index (L/min/m^2^)	2.99 ± 0.60	3.18 ± 0.69	2.85 ± 0.44	2.97 ± 0.65	0.11
MWE (mmHg L/g/min)	3.61 ± 1.68	4.82 ± 1.97	3.10 ± 1.13	2.86 ± 1.11	<0.001
LA volume index (ml/m^2^)	48 ± 16	38 ± 12	51 ± 18	54 ± 14	<0.001
LV LGE	72 (80%)	10 (37%)	32 (100%)	30 (100%)	<0.001
Native T1 (ms)	1,232 ± 119	1,193 ± 104	1,253 ± 122	1,258 ± 114	0.066
ECV, *N* = 89	46 ± 10	35 ± 5	47 ± 2	57 ± 4	<0.001
Rest MBF (ml/min/g)	0.87 ± 0.21	0.97 ± 0.19	0.85 ± 0.21	0.81 ± 0.21	0.011
Rest RPP-norm. MBF (ml/min/g)	1.00 ± 0.28	1.07 ± 0.31	0.98 ± 0.27	0.96 ± 0.26	0.3

BP, blood pressure; bpm, beats/min; ECV, extracellular volume; EF, ejection fraction; EDV, end-diastolic volume; ESV, end-systolic volume; LA, left atrial; LV, left ventricular; LGE, late gadolinium enhancement; MBF, myocardial blood flow; MWE, myocardial work efficiency; norm, normalized; RPP, rate pressure product; sys, systolic.

^a^ *n* (%); median (IQR); mean ± SD.

^b^ Fisher's exact test; Kruskal–Wallis rank sum test; Pearson's chi-squared test; one-way ANOVA.

MBF was moderately negatively correlated with LV mass index (*r* = −0.36; *p* < 0.001) and weakly negatively correlated with serum NT-proBNP (*r* = −0.23; *p* = 0.034), but was not associated with the difference in the involved and uninvolved free light-chain (dFLC) level, serum NT-proBNP, or cardiac troponin-T (Table 3).

**Table 3:** Pairwise correlations between the variables as assessed by Pearson's method.

	Rest MBF	MWE
MWE	0.42****	
ECV	−0.25*	−0.54****
Native T1	0.06	−0.15
LV ESVi	−0.51****	−0.47****
LV EDVi	−0.37***	−0.15
LV mass index	−0.36***	−0.70****
LV EF	0.49****	0.67****
LV CI	0.19	0.41****
MAP	0.13	0.47****
Troponin-T	−0.17	−0.52****
NT-proBNP	−0.23*	−0.55****
dFLC	0.08	−0.06

Pearson's coefficients: *p < 0.05; ***p < 0.001; ****p < 0.0001. CI, cardiac index; dFLC, difference in involved and uninvolved free light chains; ECV, extracellular volume; EF, ejection fraction; EDV, end-diastolic volume; ESV, end-systolic volume; LV, left ventricular; MAP, mean arterial pressure; MWE, myocardial work efficiency; NT-proBNP, N-terminal pro-B-type natriuretic peptide.

### LV function and surrogate MWE

LVEF averaged 56% ± 9% and decreased similar to MBF by ECV tertiles (Figure 2B, *p* < 0.001 for linear trend). Resting MBF was moderately correlated with LVEF (*r* = 0.49; *p* < 0.001), with a linear regression model predicting a 0.0585 ± 0.011 ml/min/g increase per 5% increment of LVEF (*p* < 0.001).

The LV mass index, LV end-systolic volume index, LV end-diastolic volume index, and peak endocardial GLS were correlated negatively with MBF (Table 3). The increasing LA volume index, a marker of diastolic dysfunction, was moderately correlated with ECV (*r* = 0.41; *p *=< 0.001).

**Figure 3:**
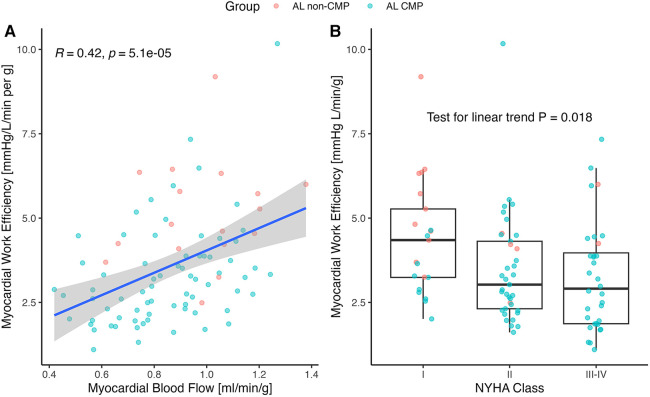
(**A**) Correlation of myocardial work efficiency (MWE) to rest myocardial blood flow (MBF) and (**B**) NYHA classification. (**A**) MBF correlated positively with the MWE, calculated as external work (mean arterial pressure×stroke volume×heart rate) divided by myocardial mass (*r *= 0.42; *p* < 0.001). The correlation in the AL-CMP group was *R* = 0.39, *p* < 0.001 with *N* = 75 patients and *R* = 0.18, *p* = 0.5 in the AL non-CMP group with *N* = 17 patients. (**B**) MWE decreased with the NYHA class. NYHA, New York Heart Association Functional Classification of Heart Failure.

Surrogate MWE averaged 3.61 ± 1.68 mmHg L/g/min and decreased significantly by ECV tertile (*p* < 0.001 for linear trend; Figure 2C). It was moderately correlated with MBF (*r* = 0.42; *p* < 0.001; Figure 3A) and LVEF (*r *= 0.67; *p* < 0.001). Surrogate MWE decreased by NYHA class from 4.39 ± 1.73 mmHg L/min/g for NYHA I to 3.62 ± 2.28 mmHg L/min/g for NYHA IV (*p* = 0.04 for linear trend; Figure 3B).

**Figure 4:**
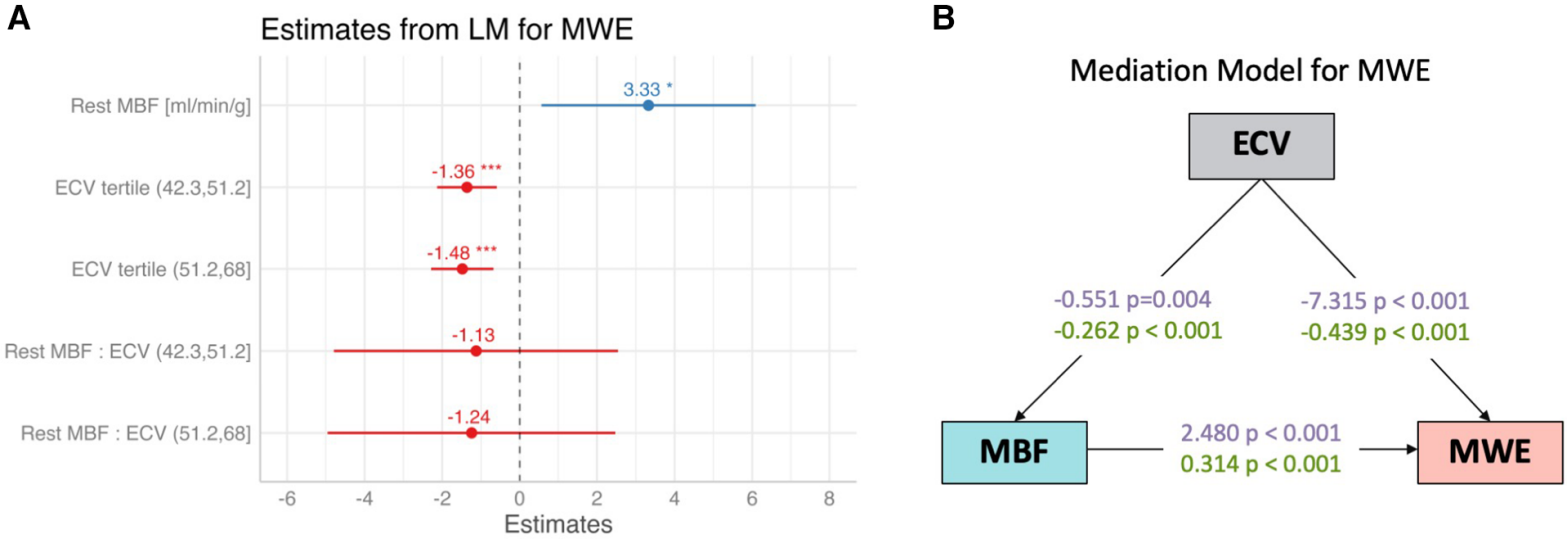
(**A**) Coefficient estimates from a multivariable linear regression model for myocardial work efficiency (MWE) with rest MBF and ECV (as tertiles) as predictors. MWE was positively associated with rest MBF, reflecting the demand–supply relationship between external cardiac work normalized by LV mass and MBF and negatively associated with ECV, a surrogate marker of amyloid burden. *p < 0.05; ***p < 0.001. (**B**) Estimates with and without normalization, with a mediation model of direct and indirect effects of extracellular volume (ECV) on myocardial work efficiency (MWE), with rest MBF (MBF) as the mediator.

In a multilinear regression model for surrogate MWE, with rest MBF and ECV as predictors, MWE was positively associated with MBF (2.48 ± 0.73; *p* = 0.001) and significantly lower for the two upper tertiles of ECV (*p* < 0.001; Figure 4A). There was no evidence of a significant interaction of MBF with ECV for the prediction of MWE. In a mediation model for surrogate MWE, ECV had significant (*p* < 0.001) direct and indirect effects on surrogate MWE, with the latter mediated by MBF, as illustrated in Figure 4B, which shows standardized coefficient estimates. Each 5% increase of ECV resulted in an indirect effect mediated by MBF amounting to a −0.068 mmHg L/min/g increase of MWE (95% CI: −0.164 to −0.017) equivalent to 16% of the total effect of ECV on surrogate MWE. The direct effect of a 5% increase of ECV on surrogate MWE was estimated to be −0.366 mmHg L/min/g (95% CI: −0.521 to −0.221; 84% of total effect). The RMSEA of the mediation model was <0.001, indicating a good fit.

### Regional variation of MBF and ECV

**Figure 5:**
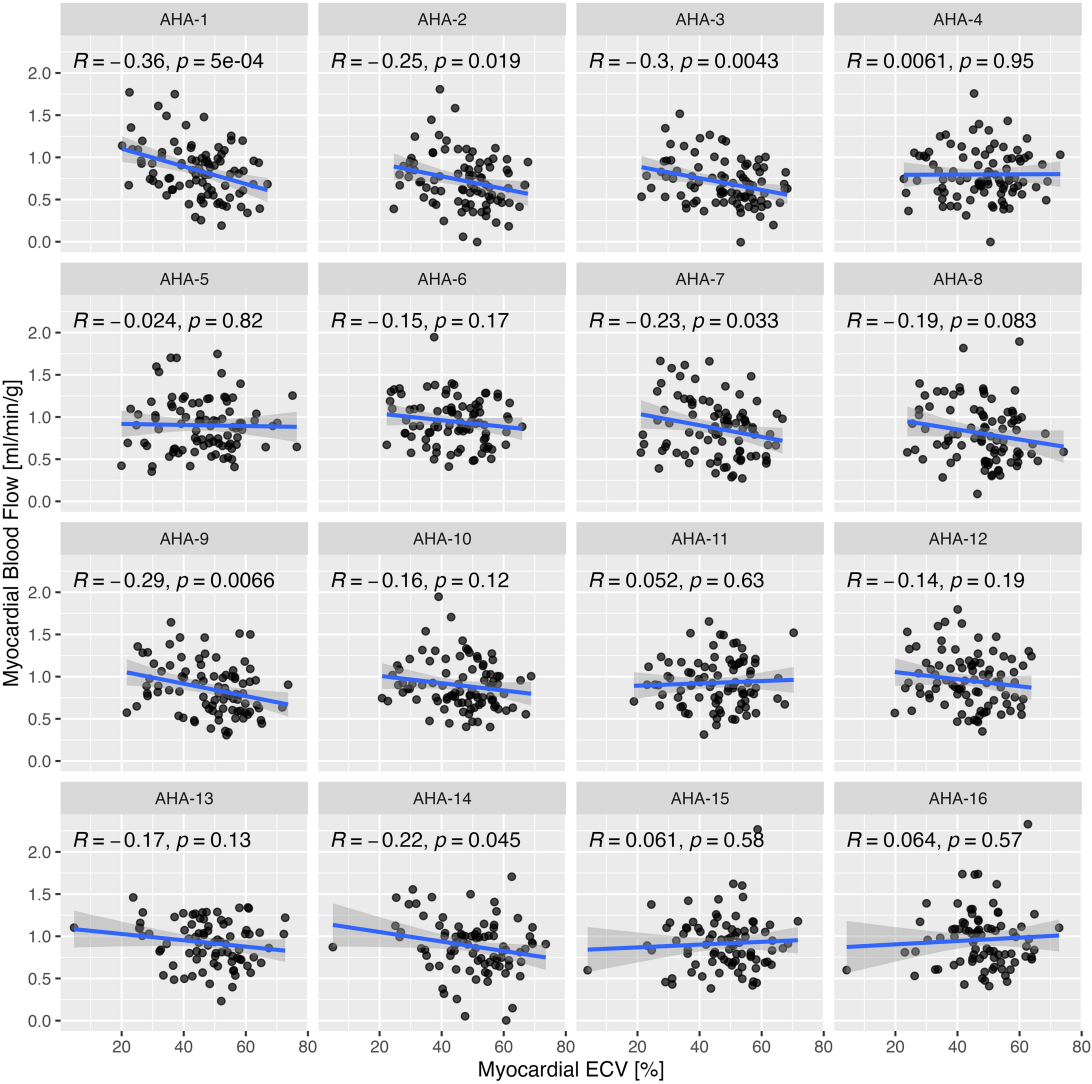
Correlation of segmental rest myocardial blood flow (MBF) with segmental ECV by myocardial segment based on the standard American Heart Association (AHA) segmentation model for LV. Correlation coefficients were calculated by Pearson's method.

**Figure 6:**
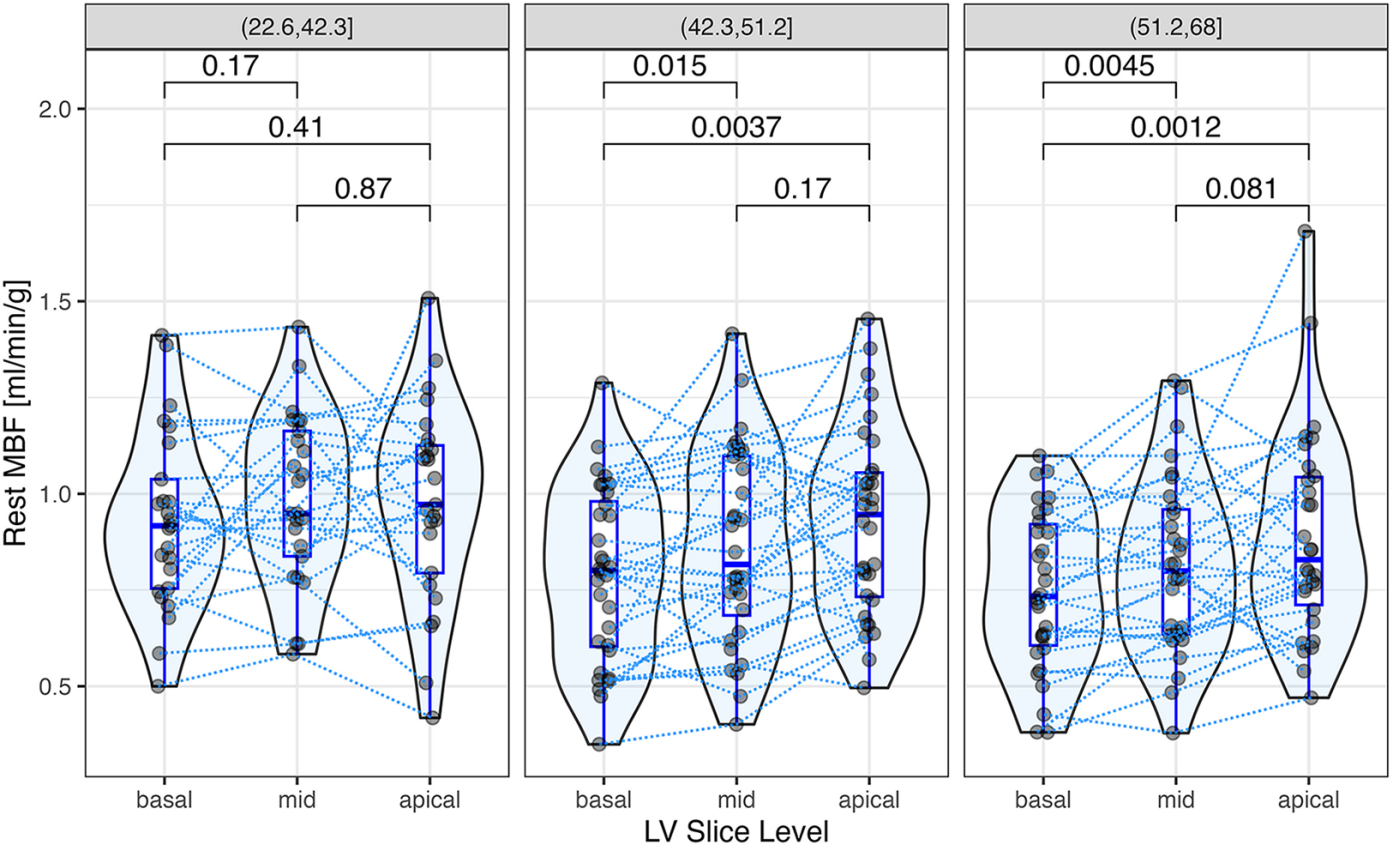
Segmental rest myocardial blood flow (MBF) at the apex, middle, and basal regions of the left ventricle. Each data point represents the average of rest MBF at the basal, middle, and apical slice levels, respectively, with data points from the same patient connected by dotted lines. Rest MBF increased significantly from the base to the apex in the two upper tertiles of ECV. The *p*-values for paired comparisons (*t*-test), shown above the brackets, were adjusted by Holm's method. A linear mixed-effects model for segmental rest MBF predicted a negative fixed effect of ECV (*p* = 0.015) and significantly higher MBFs in the middle (+0.66 ml/min/g; *p* < 0.001) and apical slices (+0.11 ml/min/g; *p* < 0.001) compared to the basal slice (0.82 ml/min/g).

Segmental MBF assessed by myocardial segment was correlated significantly with ECV in 6 of 16 segments (Figure 5). Segmental MBF adjusted by the RPP was significantly associated with segmental ECV. A linear mixed-effects model predicted a 0.003 ± 0.0012 (*p* = 0.006) decrease of rest MBF for a 1% change of ECV with simultaneous adjustment by the rate pressure product (*p* < 0.001). Segmental MBF was analyzed to assess the base-to-apex gradient. The variation of MBF from the basal to the apical slices is illustrated in Figure 6 for each tertile of ECV. One patient was found to have LGE consistent with apical scarring from ischemic disease and was excluded from this analysis. MBF averages for each slice level increased significantly from the base to the apex in the two upper tertiles of ECV. MBF was negatively associated with ECV on a per-segment basis (*p* = 0.015) and significantly higher in the middle (+0.66 ml/min/g; *p* < 0.001) and apical slices (+0.11 ml/min/g; *p* < 0.001) compared to that in the basal slice (0.82 ml/min/g).

### LGE

**Figure 7:**
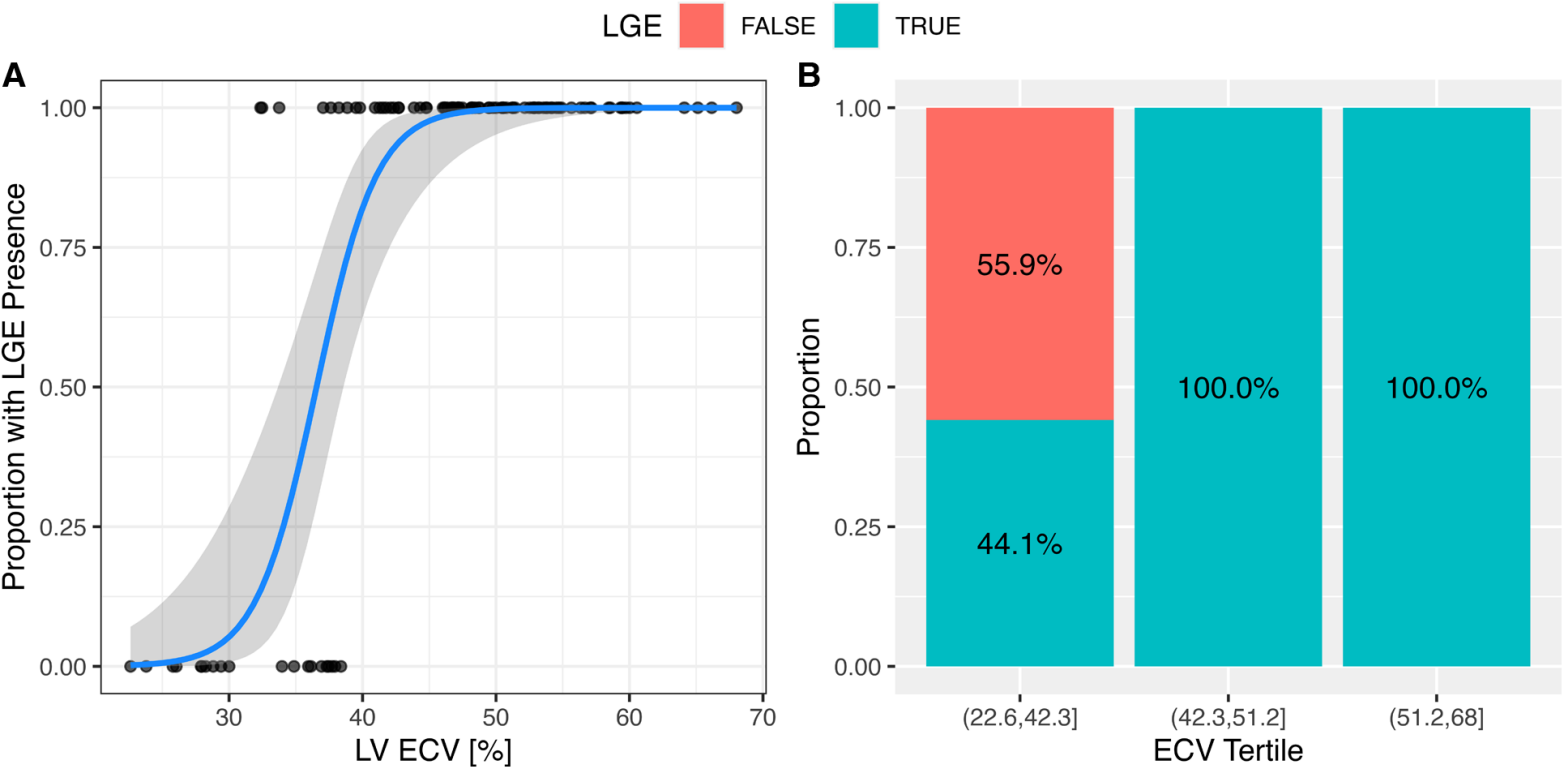
The presence of LGE was associated with the extracellular volume. (**A**) The presence of LGE in the LV was associated with global ECV. In a logistical regression model for LV LGE, each 1% change of ECV increased the odds of LV LGE by 1.55 (95% CI: 1.3–2.1; *p* < 0.001). The solid line represents the logistical regression curve estimate. (**B**) The proportion of patients with LV LGE increased by ECV tertile (*p* < 0.001; chi-squared test).

LGE in the LV was observed in 72 (80%) AL patients. The presence of LV LGE was associated with global ECV (Figure 7).

## Discussion

This study yielded several key findings about LV systolic dysfunction in subjects with AL amyloidosis. Increasing AL amyloid burden estimated by ECV was associated with LV systolic dysfunction, as assessed by LVEF and surrogate MWE. A decrease of rest MBF in AL amyloidosis is associated with higher LV ECV, an imaging biomarker related to amyloid burden and adverse extracellular matrix expansion. The role of amyloid burden and ECV expansion on surrogate MWE in AL amyloidosis has remained unclear until now, possibly because the assessment of MWE and ECV expansion was performed with different imaging modalities. Using a simple, CMR-based surrogate metric of MWE that did not require the measurement of myocardial energetics, we showed that expanded ECV as well as lower rest MBF were associated with lower surrogate MWE.

The correlation of surrogate MWE and rest MBF reflects the dependence of both quantities on EW. For surrogate MWE, the dependence is implicit in the definition of MWE as EW, normalized by LV mass. In the case of MBF, it reflects the balance between oxygen demand and cardiac workload or the proportionality of rest MBF with the RPP, an index of EW. Surrogate MWE with adjustment by MBF remained significantly lower in AL amyloidosis patients with ECV in its upper two tertiles. This finding suggests that surrogate MWE is limited by both MBF on account of a lower rest MBF in AL amyloidosis and an additional reduction of surrogate MWE is attributable to the myocardial amyloid burden for which ECV can be considered a surrogate biomarker. As the lower rest MBF in AL amyloidosis may also reflect amyloid burden (e.g., through a reduction of capillary density by interstitial space expansion with amyloid deposition), we hypothesized that ECV has a direct effect on surrogate MWE (e.g., through cardiotoxicity) and an indirect effect mediated by rest MBF. In the mediation model, the effect of ECV on MBF is negative (i.e., ECV expansion reduces rest MBF), while the association of MBF with surrogate MWE is positive (i.e., higher MWE requires higher MBF). The results of this analysis are consistent with the known pathophysiological characteristics of AL amyloidosis that can impair MWE, such as a light-chain toxicity that leads to cellular and mitochondrial dysfunction (9, 17), increased metabolic demand (17), and increased myocardial stiffness (18). A previous study by Clemmensen et al. (6) established that myocardial energy or work efficiency was reduced in AL amyloidosis and correlated with MBF. The present study confirms the association of surrogate MWE with MBF and additionally establishes a link of both surrogate MWE and MBF with ECV, a widely used marker of myocardial amyloid burden with the potential to guide amyloid-reducing treatments (19).

Although established as a non-invasive method for the assessment of myocardial external efficiency, the need for a cyclotron limits the feasibility of 11C-acetate PET/CT. We used a simple surrogate MWE measure derived from CMR in AL amyloidosis. Similarly, other investigators have used echocardiography-derived surrogates of MWE in mitral regurgitation (20) or MRI-derived surrogate myocardial power and surrogate power efficiency in aortic stenosis (21). Using a CMR-derived surrogate MWE assumes a stable myocardial oxygen consumption per gram of myocardial tissue, but cannot account for differences in amyloid burden, as amyloid will not contribute to myocardial work (22). This is an intrinsic challenge in LV mass-based estimates of myocardial external efficiency in infiltrative cardiomyopathies, as it is not feasible to completely delineate normal myocardium from amyloid burden by CMR. We chose the abovementioned method as it has been shown to be an alternative to the ^11^C-acetate-based measure of myocardial external efficiency in the cardiac amyloidosis population.

Lower rest MBF in proportion to ECV has been observed in other non-ischemic cardiomyopathies (23), suggesting that myocardial interstitial remodeling manifested by ECV expansion can result in reduced MBF. A distinguishing feature of AL amyloidosis is that in addition to effects from structural and vascular remodeling, there may also be cytotoxic effects from circulating light chains (9), which may have a deleterious impact on LV systolic function due to cardiomyocyte injury. This light-chain toxicity is likely to manifest through lower surrogate MWE and systolic dysfunction and a reduced capacity to increase cardiac workload. Our prior study (7) and a recent study investigated myocardial ischemia in a cohort of patients with cardiac amyloidosis (including AL and transthyretin, ATTR, cardiac amyloidosis) and control patients (24). MWE was not evaluated in these studies. The current study adds to prior studies and implicates increasing amyloid burden as a potential cause of impaired MBF and surrogate MWE at rest, furthering our understanding of cardiac dysfunction in this disease.

In AL amyloidosis, an elevated ECV may reflect a combination of fibrosis and amyloid deposition. Notably, we saw an abnormally low MBF in the remission subjects, consistent with the observation by Cuddy et al. (25) that ECV remains elevated in the remission subgroup despite a successful reduction of dFLC levels. Diffuse myocardial fibrosis can coexist with amyloidosis (26), lower myocardial efficiency (6), and negatively impact systolic performance, but it is not possible to distinguish fibrosis from amyloid deposition using current MRI-based methods.

The analysis of segmental MBF allowed the determination of the effects of ECV on MBF independently of its global effect on systolic pump performance. Segmental MBF was associated with the variation of ECV within the heart. It also showed a positive base-to-apex gradient in the two upper tertiles of ECV, a finding consistent with apical sparing observed in previous studies of cardiac amyloidosis (27). The reason that the cardiac apex is less affected by systolic dysfunction in subjects with AL amyloidosis remains unclear, but this pattern is opposite to the negative base-to-apex MBF gradients observed in subjects with coronary risk factors and/or coronary heart disease (28, 29).

### Limitations

This study has several limitations. First, the study size may have been inadequate to fully reveal the effects of cardiac AL amyloid deposition on rest MBF. The study size was modest due to the rarity of AL amyloidosis as well as our stringent inclusion and exclusion criteria. Second, the recent AL amyloidosis and AL remission subjects are separate and do not represent a longitudinal follow-up. When evaluating these subjects in AL remission, a degree of selection bias may be anticipated as healthier subjects with less cardiac involvement would presumably be more likely to survive until follow-up. Further studies assessing longitudinal follow-up will be important to help clarify the effects of disease progression and treatment on surrogate MWE. Our cohort included individuals with documented AL amyloidosis and did not include control cohorts of healthy subjects or those with other etiologies of ventricular dysfunction for comparison. This study utilized institutional cutoffs for troponin-T and NT-proBNP and not specific cutoffs validated for cardiac amyloidosis. However, as our primary analysis stratified patients by ECV textiles, a validated measure of cardiac amyloidosis burden, the findings of this study are not affected by the biomarker cutoffs. We cannot exclude the possibility that contamination of segmental signal intensity averages from the blood pool could lead to a positive bias to overestimate blood flow, particularly in segments with thinner walls or near the apex, which is susceptible to partial volume effects. Nevertheless, a base-to-apex gradient for MBF was observed for the two higher tertiles of ECV for which the segmental wall thickness was larger; therefore, the likelihood of contamination from the blood pool is lower. We did not assess stress perfusion by MRI to reduce the subject testing burden. Although patients with known obstructive epicardial coronary disease were excluded, not all patients underwent left heart catheterization or CT coronary angiography; therefore, the prevalence of coronary artery disease may have been underappreciated. However, as noted by Chacko et al., rest MBF was significantly elevated in patients with both obstructive and non-obstructive coronary artery diseases when compared to the CA population (16). Therefore, this underappreciation is unlikely to significantly affect our findings of the effects of rest MBF and ECV on surrogate MWE.

## Conclusions

In this study, high AL amyloid burden was related to reduced rest MBF, reduced LV systolic function, and lower surrogate MWE. The adverse structural and vascular changes from amyloid, which expand the ECV and impair perfusion, appear to contribute to lower MWE in AL amyloidosis. Rest MBF remains depressed after successful therapy of AL amyloidosis and hematological remission, consistent with the persistent interstitial remodeling from amyloid and fibrosis.

## Data Availability

The raw data supporting the conclusions of this article will be made available by the authors, without undue reservation.
